# Treatment of Deciduous Teeth

**Published:** 1875-08

**Authors:** Henry S. Chase


### SELECTED ARTICLES.

### ARTICLE IV.

### *Treatment of Deciduous Teeth.*

BY HENRY S. CHASE.

There is nothing more worthy of attention, at the present time, by the dental profession, than the subject of *the Preservation of the Decidnous Teeth.*

That we should go to the *source* of the trouble, and try to understand the remote causes of cecay in these temporary organs, and thereby endeavor to institue measures for prevention of dental caries, hardly any one will deny. As I have already on other occasions written lengthy papers on this subject, one of which was read at the Amerieau Dental Association in Boston, in 1866, and which I intend to reproduce in this Journal soon, I will pass this portion of the subject and proceed to make some observations on the subject at the head of this article.

And in the first place it seems necessary that I should say something in regard to its importance, for until it is conceded important by dentists themselves, it will grow into importance very slowly in the minds of fathers and mothers. It certainly is true that the public has been educated up to the present point, in dental matters, by dental practitioners themselves. And on this particular subject a great deal of preaching is still necessary. For while a large number of families among the well-to-do of our cities have their children's temporary teeth attended to, the great mass, consisting of nineteen-twentieths, regard it as of no consequence. And the result is a very great deal of dental pain endured by children, and a great loss of sleep and waste of sympathy by parents. I wish that every parent was obliged by natural law to suffer all the pain endured by children, resulting from decay of their deciduous teeth. I think it would only be just. It is the parents' duty to give by inheritance good, sound dentos to their children, and it is a further duty that they enforce such hygienic and remedial measures as will preserve them until nature calls for their removal.

These deciduous teeth are necessary for the health of the child. Food not well masticated is imperfectly digested, resulting in many of the ailments of childhood, including eot only "bowel troubles," but many diseases resulting from want of proper nutrition, such as nervous disorders, rachitis, imbecility and starvation of the dental tissues

Not only is this the case, but the ill effects of pain on the whole organism, together with the inflammation of the parts contiguous to dental disease, frequently produce the most disastrous consequences.

That the presence of healthy deciduous teeth in the jaws until the natural development of the permanent set, is necessary for the natural evolution of the jaws and ieeth, I have no doubt. That deciduous teeth may be extracted before time, and yet a good development of these organs take place in many instances, I also believe. But on the other hand, I can see an arrest of development produced by premature extraction.

The growth of the jaws takes place in several different ways, in order to give room for the permanent teeth. *One* of its modes of growth is by a movement of the temporary crown bodily forward toward the buccal or lingual surface. Not until the roots of the deciduous teeth have been more or less resorbed can this take place ; then the alveolar wall above the crown of the deciduous tooth is wholly or partially resorbed, and the permanent crowns moves toward the buccal or ligual surface, pushing the gum before it also. When the tooth has attained the desired position, the alveolar wall is again formed under the gum, and thus an increase of transverse and antero-posterior diameter of the arch takes place.

The other modes by which this result has been attained need not at this time be discussed. Every dentist of many years experience and observation must have often noticed permanent teeth coming through the gum above the crowns of the temporary, showing, not the cutting edge or grinding border first, but the broad buccal or lingual face. When the temporary tooth is absent for a considerable period before the eruption of the permanent substitute, I have never seen the latter take the position described, but on the contrary, it shows its *edge* first, and then in the line representing the smaller arch.

This tendency of the permanent teeth, under the condition of premature extraction of the deciduous ones, may be taken advantage of in those cases when there is *too much* fullness of the dental arch, and especially of its anterior portion.

And now that, I think, I have given sufficient reasons for the preservation of these deciduous teeth, let us find out the best way to do it. I shall give the results of *my own experience*, and *others have the same privilegc in this Journal.*

Twenty years ago I commenced the practice of endeavoring to impress on the minds of parents the importance of this subject. Children three years old and upwards, used to be brought to have teeth extracted on account of toothache. I began to save them instead of extracting. I soon found that it was a matter that must be studied. In the first place I must get the confidence of tee little ones, avoiding a movement which would give it pain. I must never deceive them. I must not fatigue them. To do this I found much patience was required. I found that it was not a desirable practice to have, so far as money and comfort were concerned. Nothing but a sense of duty and sympathy for the parties urged me on. I met with more difficulties then than now, and I do not intend to fatigue my reader with the process of evolution, but tell them what my *present* practice is, being as it is. the result of my many mistakes and successes by diverse methods.

In the first place, what deciduous teeth do I not try to save, but on the contrary extract?

I extract all deed roots, whether with or without crowns. Now right here, let me repeat what I have thousands of times before said. A tooth is not necessarily dead because its pulp is dead. On the contrary, a tooth with a dead pulp almost always has a living pericementum, for a long time after the death of the former. I extract all teeth having pericementitis, which I think I cannot easily cure. It is bad practice to allow roots which are causing inflammation to remain contiguous to undeveloped teeth. If a child comes to me for the first time with teeth that need extracting, and also those which need plugging, I perform the latter operation first, if possible, so as to produce a less unpleasant impression than by a different procedure. When we once get the confidence of the child by having performed

*painless operations*, then it will not be destroyed by a painful one, if we do not *deceive* the child. When the latter asks me "will it hurt?" I always tell it just what I honestly believe. It cannot be too thoroughly impressed on the minds of parents, that children ought to be brought to have their teeth filled *before they have ached.*

If a child is brought to me at four years of age for consultation, and ever after that as often as I may designate, that child need never have the toothache, or the parents wakeful nights on that account. This as a rule. There are children whom I cannot manage, but they are few, if the parents will not *meddle.*

Painless operations must be performed. Fatigue of the child must be also avoided. Give it short sittings, say from fifteen minutes to half an hour, according to age and endurance. Even a quarter of an hour may be too long. What time is lost in brevity must be made up by freqeency. I cannot insist too much on these things. Parents will urge longer sittings, because it is trouble to themselves, but be firm and look only to the success of your treatment. When these fundamental principles are well *felt* by the dentist, his practice with the children will glide into natural channels, without too much particularizing on my part.

I find that operations performed on children's teeth, are not as successful as a rule, as upon those of adults. One reason is the necessarily imperfect manipulations in many cases. The very fact that pain must be avoided makes it so. On the other hand, deciduous teeth are full of vitality, of motion; the microscopical organs composing them are active; if a plug is not in harmony with its walls, resorption may take place and the pulp become irritated or *exposed.* It is well to remove superficial decay when possible; especially on proximate surfaces the teeth should be cut away freely, and always so as to be as self-cleansing as possible. This is still more important when one proximate surface is a sixth year molar or bicuspid; the deciduous tooth should be far removed. When plugs are placed on proximate surfaces the same separations should be maintained, for plugs and teeth will last much longer than if the natural shape of the teeth is maintained, or a *narrow* space left between the teeth. Some grinding surface must be sacrificed for the greater good of durability. Separations are easier performed for children with Arthur's thin chisels, than with the file or corundum wheel. Experience with each individual child will decide this.

In some cases alveolar abscess may be cured for children, not by the heroic treatment of adults, but by merely cleansing the cavity of decay ond pulp cavity as thoroughly as possible, using alcohol as a disinfectant. Filling the roots must not be insisted on, and attempts in this direction carried only so far as circumstances may dictate. The roots may be half absorbed in their length when the tooth is treated, or even cut half way off near the neck by the same process. When not more than a year will probably elapse before the teeth of replacement will appear, an ulcerated tooth had better be extracted.

The pulp, when exposed, may be destroyed in the usual manner, and its extirpation postponed for twelve days, in order to avoid pain, and even longer if necessary. No attempt should be made to *dive* into the root canals for the vessels; saturate them well with alcohol and cover them up. The rubber dam must often be dispensed with, and pieces of spunk cut into large or small squares to absorb moisture must be freely used.

The pulp chamber in deciduous teeth is very large, often occupying a large portion of the crown *above the neck*, with horns running towards each cusp. Great care must be used not to wound this pulp or its horns in excavating, for they are exposed in very many small crown cavities, and especially is this the case when the resorptive process has commenced. When freshly exposed, or not having had many inflammations, efforts should be made to preserve it alive. To do this ox. chlo. zinc must not come in contact with the pulp or with dentine. Ox. chlo. zinc is not in harmony with the delicate tissues of deciduous teeth.

Gutta-percha varnish, (chloroform and gutta-percha in thick solntion) that will drop from an instrument, should always be used for an exposed pulp, it will quiet the pain and keep the air from the pulp; drying as it does, rapidly, the plug can be introduced immediately, either of ox. chlo. zinc or metallic paste. For a temporary filling the former may be used, as it hardens rapidly, and can be introduced with little pressure. But it is much better to *fill the cavity but once.* It will suit parent and child much better than another operation. The thorough removal of decay must not be insisted on when accompanied with pain. Its removal is desirable and should be done when it will not prevent the accomplishment of our object, but "a half loaf is better than no bread," and therefore there may be cases when but little of the decay can be removed. Saturation of the carious bone with alcohol will render it less liable to decay, and if the margins of the cavity are cut away until sound and healthy dentors is reached, decay will proceed very slowly under a water-tight plug. The metallic paste should be the best amalgam that can be procured. I have found "Fletcher's Gold and Platinum Alloy" to be the most reliable. It neither shrinks or discolors, and hardens readily enough, and bears mastication well. I have tried this by the "glass tube test" many times, and never found leakage. Sometimes it must be used very plastic, at other times more friable; but it must be packed soon after mixing. Every cavity in which this paste is used should be *varnished.* This coating has less conducting power than the metals, and seems to be more in harmony with deciduous dentos. The patient should return in a day or two for examination. The plugs may have been partially displaced by mastication, notwithstanding your directions that the tooth should not be used, in eating, for four hours. At this time it should also be polished, if necessary. I never had but one such plug spoiled by a patient.—*Missouri Dental Journal.*

